# Global Prevalence of Q Fever in Camels: A Systematic Review and Meta‐Analysis of Studies From Africa and Asia

**DOI:** 10.1002/vms3.71275

**Published:** 2026-10-21

**Authors:** Simegnew Adugna Kallu, Shimels Mengistu, Pawlos Wasihun Asnake, Ambachew Motbaynor Wubaye, Migbaru Keffale Bezabih, Melkamu Temesgen Mekonen, Sindu Zegeye Alamneh, Yusuf Ahmed Elmi, Kedir Sali, Simachew Getaneh Endalamew, Amsalu Misgie Molla, Yihenew Getahun Ambaw

**Affiliations:** ^1^ College of Veterinary Medicine Haramaya University Dire Dawa Ethiopia; ^2^ Department of Veterinary Science Burie Campus Debre Markos University Burie Ethiopia; ^3^ Department of Veterinary Science, College of Agriculture and Environmental Sciences Debre Tabor University Debre Tabor Ethiopia; ^4^ Armauer Hansen Research Institute Addis Ababa Ethiopia; ^5^ Department of Biotechnology, College of Computational and Natural Sciences Debre Markos University Debre Markos Ethiopia; ^6^ Department of Veterinary Epidemiology and Public Health, School of Veterinary Medicine Bahir Dar University Bahir Dar Ethiopia; ^7^ Department of Veterinary Medicine, College of Agricultural Sciences Woldia University Weldiya Ethiopia

**Keywords:** *C. burnetii*, camels, prevalence, Q fever

## Abstract

Q fever is a neglected zoonotic disease caused by *Coxiella burnetii* with a global distribution. It has a significant public health implication in resource‐limited settings. Therefore, this systematic review and meta‐analysis was designed to comprehensively analyse the global prevalence of Q fever in camels. Following the Preferred Reporting Items for Systematic Reviews and Meta‐Analyses (PRISMA) guidelines, online searches were conducted in Pubmed, ScienceDirect, Scopus, Web of Science and Google Scholar from 9 to 20 December 2025, to retrieve articles published between 1 January 2000 and 30 November 2025. After excluding articles that did not meet the inclusion criteria, this systematic review and meta‐analysis identified 35 articles. After heterogeneity assessment, random effects model was used to estimate the pooled prevalence, and the overall pooled prevalence of Q fever in camels was 33.37% (95% CI: [23.14; 45.44]), combining serological evidence of previous or recent exposure with molecular evidence of *C. burnetii* detection. Diagnostic‐method‐specific analyses showed substantially different pooled estimates for ELISA (44.06%) and PCR (7.76%), reflecting distinct biological endpoints. The prevalence of Q fever was 37.17% in Africa and 26.87% in Asia. This meta‐analysis result showed that the prevalence of Q fever is high in camels, but it is still neglected in several regions, posing significant impact to human and animal health. Expanded epidemiological investigations to underrepresented geographic regions and production systems will provide critical evidence to inform global surveillance strategies and support evidence‐based policy for zoonotic disease prevention in camel populations and beyond.

## Graphical abstract

Global Q Fever in Camels: A Systematic Review and Meta‐analysis

- Evidence: 35 studies (2000–2025)
- Global pooled prevalence: 33.37% (95% CI: 23.14–45.44%)
- ELISA seroprevalence: 44.06%
- PCR molecular prevalence: 7.76%
- Africa: 37.17%
- Asia: 26.87%

Conclusion: Q fever is an important, under‐recognized One Health concern. Expanded surveillance in underrepresented regions and camel production systems is needed to guide zoonotic disease prevention.

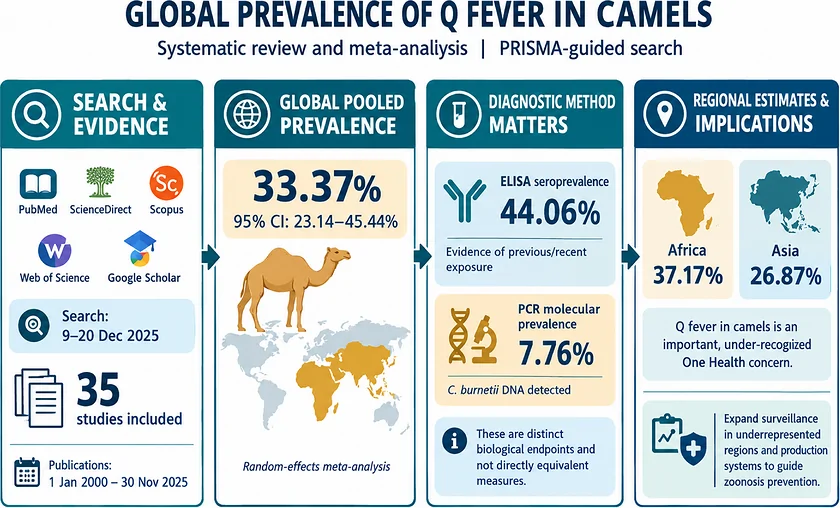

## 1 Introduction

Q fever or Coxiellosis is a neglected zoonotic disease caused by the obligate intracellular, Gram‑negative bacterium *Coxiella burnetii* (*C. burnetii*) (Hechemy 2012). This pathogen has a worldwide distribution with an exceptionally broad host range that includes humans, domestic livestock and pets, wildlife, reptiles, fish, birds and ticks (Cutler et al. 2007; WOAH 2019). Domestic ruminants particularly cattle, goats, sheep and camels constitute the principal reservoirs and sources of human infection (Candela et al. 2017; Devaux et al. 2020; Pexara et al. 2018).

*C. burnetii* is known for its high infectious capacity, with as few as 1–10 organisms able to establish an infection in humans through the inhalation of contaminated aerosols (Eldin et al. 2017). This bacterium is released in substantial concentrations through the placenta, foetal membranes, amniotic fluid, milk, urine and faeces of infected animals, making periparturient animals major sources of transmission (Angelakis and Raoult 2010). Moreover, *C. burnetii* possesses the ability to form spore‐like structures, allowing it to persist in the environment for extended periods, thereby enhancing its epidemiological importance (Kersh et al. 2013).

Camels, particularly dromedary camels (*Camelus dromedarius*), are widely distributed across arid and semi‐arid regions of Africa, Asia and the Middle East, where they serve as important livestock for pastoral and agropastoral communities. The close contact between human herders and infected camels, combined with the high infectivity of *C. burnetii*, places pastoral and nomadic populations at substantial risk of infection. The first documented case of Q fever in camels was reported in the 1950s (Kaplan and Bertagna 1955), establishing dromedary camels as a recognized reservoir for *C. burnetii* infection. Several cross‐sectional serological surveys have documented the presence of *C. burnetii* seropositivity in camels across Africa (Klemmer et al. 2018; Larson et al. 2019; Milkesa et al. 2024; Rooney et al. 2024; Selmi et al. 2018) and Asia (Holloway et al. 2023; Hussain et al. 2022; Kasem et al. 2025; Sheek‐Hussein et al. 2025), suggesting endemic circulation of the pathogen in camel populations. However, the true burden of disease remains poorly characterized due to limited surveillance data and the scarcity of comprehensive epidemiological studies in these regions.

Understanding the prevalence and geographic distribution of Q fever in camels is critical for informing public health surveillance, risk assessment and prevention strategies in vulnerable populations. Despite the importance of this zoonosis and numerous studies reporting the prevalence of Q fever in camels from different countries, the findings are highly variable because of differences in geographical settings, diagnostic methods and study designs. To our knowledge, no comprehensive global systematic review and meta‐analysis, specifically focused on camels, has synthesized these data to provide an overall prevalence estimate and evaluate sources of heterogeneity. Therefore, this systematic review and meta‐analysis aimed to estimate the global prevalence of Q fever in camels and to identify potential sources of heterogeneity across different diagnostic methods and geographic regions.

## 2 Material and Methods

### 2.1 Study Setting

The Preferred Reporting Items for Systematic Reviews and Meta‐Analyses (PRISMA) 2020 checklist was followed to guide study identification, selection, critical appraisal, synthesis and reporting of this evidence‐based systematic review and meta‐analysis (Page et al. 2021). The condition, context, population (CoCoPo) framework was additionally applied to structure the search strategy and ensure comprehensive yet focused inclusion of relevant studies (Munn et al. 2015).

### 2.2 Literature Search Strategy and Selection Process

Studies conducted on the prevalence of Q fever in camels were searched in major databases (PubMed, Scopus, Web of Science, Science Direct, Google Scholar) using advanced search options. The search was conducted using the keywords ([‘Q fever’ OR ‘*Coxiella burnetii*’ OR ‘*C. burnetii*’ OR ‘Coxiellosis’] AND [‘prevalence’ OR ‘seroprevalence’ OR ‘serosurvey’ OR ‘epidemiology’ OR ‘infection’] AND [‘camels’ OR ‘dromedary’ OR ‘livestock’ OR ‘animals’]). All relevant published articles on Q fever in camels between 1 January 2000 and 30 November 2025 were retrieved, and the search was done from 9 to 20 December 2025.

### 2.3 Eligibility Criteria and Data Extraction

Eligible published articles were selected based on predefined criteria, including studies specified for Q fever, published between 1 January 2000 and 30 November 2025, specified total number of camels sampled, total number of camels tested positive and described the diagnostic method used.

The selection process was conducted based on the PRISMA flowchart, showing the total number of studies retrieved, those included in this study and excluded from analysis with reason of exclusion (Figure 1). Two independent teams (**MTM** and **YAE**; **AMW** and **AMM**) screened the studies based on their title and abstract and further assessed the full text of the studies based on the above criteria to decide on the inclusion and exclusion of the studies. Disagreements in the inclusion and exclusion of articles were resolved through discussion and consensus.

**FIGURE 1:**
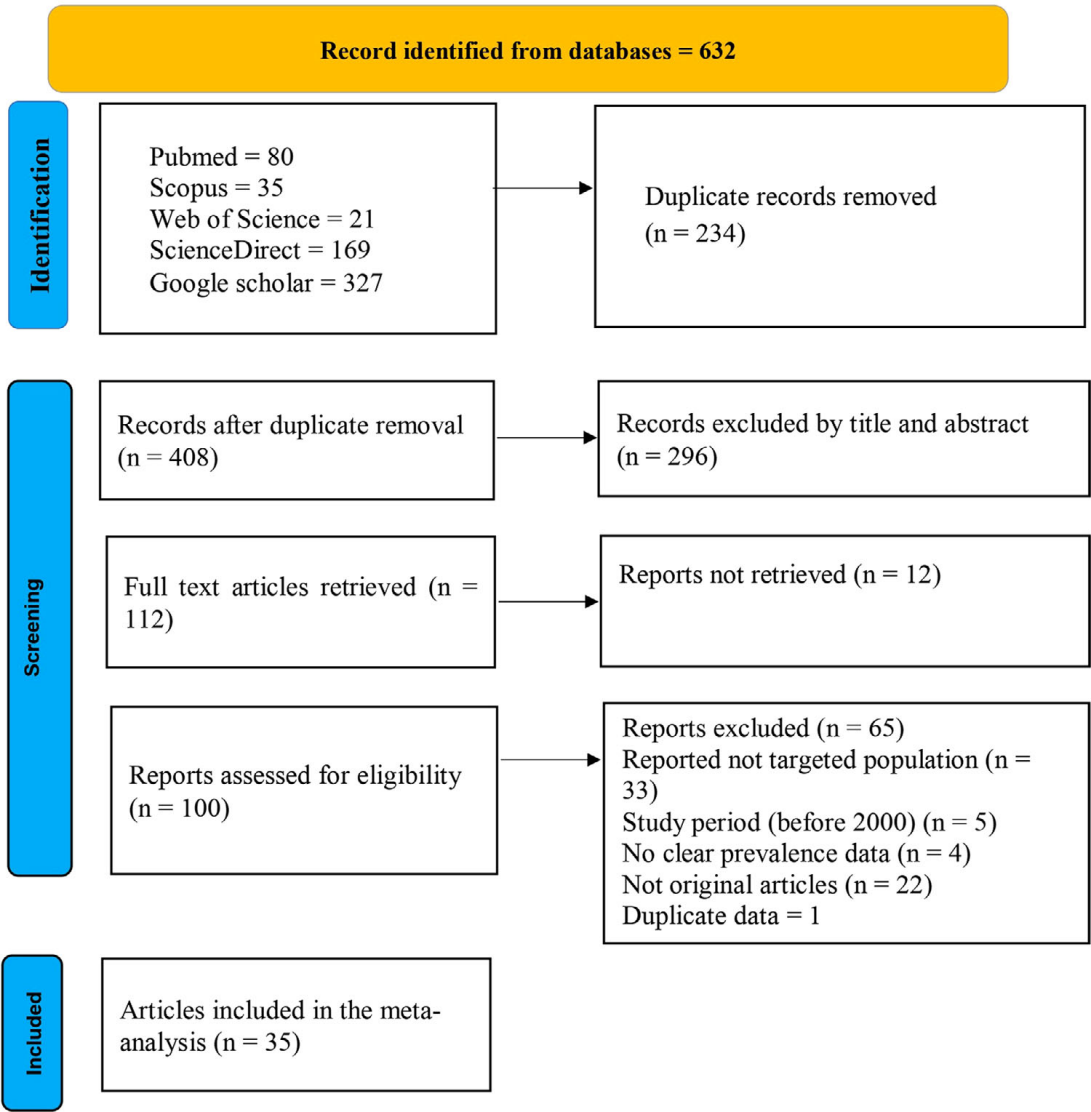
PRISMA flow diagram of the selection of eligible studies.

After excluding articles that do not met the inclusion criteria, 35 articles were selected for the analysis. Two authors (**SM** and **YGA**) independently extracted data using a standardized excel format including title, authors, year of publication, country of study, diagnostic method, number of camels tested and number of camels tested positive.

### 2.4 Quality Assessment

The quality of eligible studies included in this systematic review and meta‐analysis was evaluated by three reviewers (**PWA, KS** and **SZA**) using the Joanna Briggs Institute (JBI) critical appraisal tool for the prevalence studies (Munn et al. 2015). This tool consists of nine items that assess the internal validity and potential sources of bias and each item was scored as ‘Yes’, ‘No’, ‘Unclear’ or ‘Not Applicable’ (Table S1). Any disagreements between the two reviewers were resolved through discussion. Quality scores were calculated by awarding 1 point for each ‘Yes’ or ‘Not applicable’ response and 0 points for each ‘No’, ‘Unclear’ or missing response across the nine JBI items. Studies were then categorized by their total score as high quality (7–9 points), moderate quality (4–6 points) or low quality (0–3 points) (Table 1). All studies included in this systematic review and meta‐analysis achieved moderate to high methodological quality (4–9 points), so none were excluded due to inadequate design. Consequently, the overall evidence base was deemed sufficiently robust to justify quantitative synthesis.

**TABLE 1:** Included studies in the analysis of *C. burnetii* prevalence in camels.

Authors	Number tested	Number positive	Prevalence (95% CI)	Country	Detection method	Quality score
Holloway et al. (2023)	357	189	52.94 [47.62; 58.20]	Jordan	ELISA	9
Milkesa et al. (2024)	736	209	28.40 [25.19; 31.83]	Ethiopia	ELISA	9
Schelling et al. (2003)	142	114	80.28 [72.59; 86.29]	Chad	ELISA	6
Heidari et al. (2023)	113	7	6.19 [2.74; 12.80]	Iran	PCR	7
Bellabidi et al. (2020)	184	138	75.0 [67.99; 80.95]	Algeria	ELISA	7
Selmi et al. (2018)	534	237	44.38 [40.13; 48.72]	Tunisia	ELISA	7
Rooney et al. (2024)	233	122	52.36 [45.75; 58.89]	Kenya	ELISA	7
Kidambasi et al. (2020)	249	0	0 [0.0; 1.89]	Kenya	PCR	9
Muema et al. (2022)	189	73	38.62 [31.72; 45.99]	Kenya	ELISA	9
Al‐Graibawi et al. (2021)	91	18	19.78 [12.45; 29.72]	Iraq	ELISA	7
Doosti et al. (2014)	130	14	10.77 [6.23; 17.72]	Iran	PCR	6
Abdullah et al. (2018)	113	52	46.02 [36.69; 55.62]	Egypt	PCR	8
Ashour et al. (2024)	133	21	15.79 [10.26; 23.36]	Egypt	PCR	8
Bessas et al. (2022)	80	0	0.0 [0.0; 5.71]	Algeria	PCR	7
Bahari et al. (2021)	100	0	0.0 [0.0; 4.61]	Iran	PCR	8
Kasem et al. (2025)	2172	1014	46.69 [44.57; 48.81]	Saudi Arabia	ELISA	9
Sheek‐Hussein et al. (2025)	393	208	52.93 [47.86; 57.93]	United Arab Emirates	ELISA	9
Abdullah et al. (2019)	112	19	16.96 [10.78; 25.48]	Egypt	PCR	7
Klemmer et al. (2018)	528	211	39.96 [35.78; 44.29]	Egypt	ELISA	9
Mutisya et al. (2025)	97	28	28.87 [20.34; 39.09]	Kenya	ELISA	9
Janati Pirouz et al. (2015)	167	48	28.74 [22.15; 36.34]	Iran	ELISA	9
Muturi et al. (2021)	120	25	20.83 [14.18; 29.40]	Kenya	ELISA	9
Alkenani et al. (2024)	341	172	50.44 [45.01; 55.86]	Saudi Arabia	ELISA	9
Hussain et al. (2022)	920	190	20.65 [18.11; 23.44]	Pakistan	ELISA	9
Selim and Ali (2020)	315	69	21.90 [17.55; 26.96]	Egypt	ELISA	9
Benaissa et al. (2017)	184	132	71.74 [64.56; 78.12]	Algeria	ELISA	9
Oakley et al. (2024)	258	42	16.28 [12.11; 21.48]	Ethiopia	ELISA	9
Gumi et al. (2013)	90	80	88.89 [80.08; 94.25]	Ethiopia	ELISA	6
Ibrahim et al. (2021)	141	79	56.03 [47.43; 64.29]	Ethiopia	ELISA	9
Hussien et al. (2016)	76	49	64.47 [52.59; 74.88]	Sudan	ELISA	8
Browne et al. (2017)	334	62	18.56 [14.62; 23.24]	Kenya	ELISA	9
Hussein et al. (2015)	489	252	51.53 [47.01; 56.04]	Saudi Arabia	ELISA	9
Eckstein et al. (2022)	500	368	73.60 [69.46; 77.37]	Tunisia	ELISA	9
Larson et al. (2019)	312	62	19.87 [15.68; 24.83]	Kenya	ELISA	9
Ambreen et al. (2025)	300	59	19.67 [15.41; 24.71]	Pakistan	PCR	6

### 2.5 Statistical Procedures and Data Analysis

The meta‐analysis was performed using R version 4.5.2 (R Core Team 2025) and the pooled prevalence of *C. burnetii* was estimated using the meta package in Rstudio. Prevalence proportions were analysed on the logit scale to stabilize variances and better approximate normality of the effect‐size distribution. For studies reporting zero events, a continuity correction of 0.5 was applied to permit the logit transformation. To assess the robustness of the pooled estimate to the choice of transformation, a sensitivity analysis was performed using the Freeman–Tukey double‐arcsine transformation. Heterogeneity across studies was evaluated with Cochran's Q, *I* ^2^ (Higgins and Thompson 2002), *τ* ^2^ and prediction intervals (IntHout et al. 2016). A random‐effects model was applied when substantial heterogeneity was present and a forest plot was used to visualize the meta‐analysis output.

Subgroup and analysis were conducted based on continent, country, diagnostic test, year of publication and sample size category to explore potential sources of heterogeneity for the prevalence of Q fever using random‐effects model. We conducted several diagnostic and sensitivity analyses to evaluate the robustness of the meta‐analytic findings. Galbraith (radial) plots were used to examine the relationship between study precision and standardized effect sizes and to visually identify outlying studies and patterns of heterogeneity. Baujat plots were applied to pinpoint studies contributing disproportionately to overall heterogeneity and the pooled effect estimate (Baujat et al. 2002). In addition, a leave‐one‐out meta‐analysis was performed, sequentially omitting each study and recalculating the pooled prevalence and heterogeneity statistics (Viechtbauer and Cheung 2010). These procedures quantified the influence of individual studies on the summary estimates and heterogeneity and thereby assessed the robustness of the results.

Publication bias was assessed using funnel plot symmetry and formal tests including Egger's weighted regression (Egger et al. 1997) and Begg's rank correlation (Begg and Mazumdar 1994) with *p*‐value < 0.05 indicating significant publication bias.

## 3 Result

### 3.1 Description of Included Studies

In our comprehensive worldwide search for prevalence studies on Q fever in camels, eligible data were exclusively identified only from Africa and Asia (Table 1). The distribution of the included studies was 65.7% from Africa and 34.3% from Asia. In Africa, *C. burnetii* prevalence in camels was reported in seven countries, including Kenya (7 studies), Egypt (5), Ethiopia (4), Algeria (3), Tunisia (2) and Chad and Sudan (1 each). In Asia, studies were identified from six countries: Iran (4), Saudi Arabia (3 studies), Pakistan (2) and United Arab Emirates, Jordan and Iraq (1 each).

### 3.2 Pooled Prevalence and Subgroup Analysis

A total of 11,233 camels were tested, of which 4363 were positive for *C. burnetii* (Figure 2; Table 2). Significant heterogeneity between studies (*Q*_34_ = 1278.56, *I* ^2^ = 97.3%, *p* < 0.0001) was detected. This strong heterogeneity was additionally evidenced with a wide predictive interval of [3.55%–87.20%] demonstrating substantial differences between study variability in the prevalence estimates. Due to this high heterogeneity, a random effects model was used to calculate the pooled prevalence. The pooled prevalence of *C. burnetii* in camels in this systematic review and meta‐analysis was 33.37% (95% CI: [23.14; 45.44]).

**FIGURE 2:**
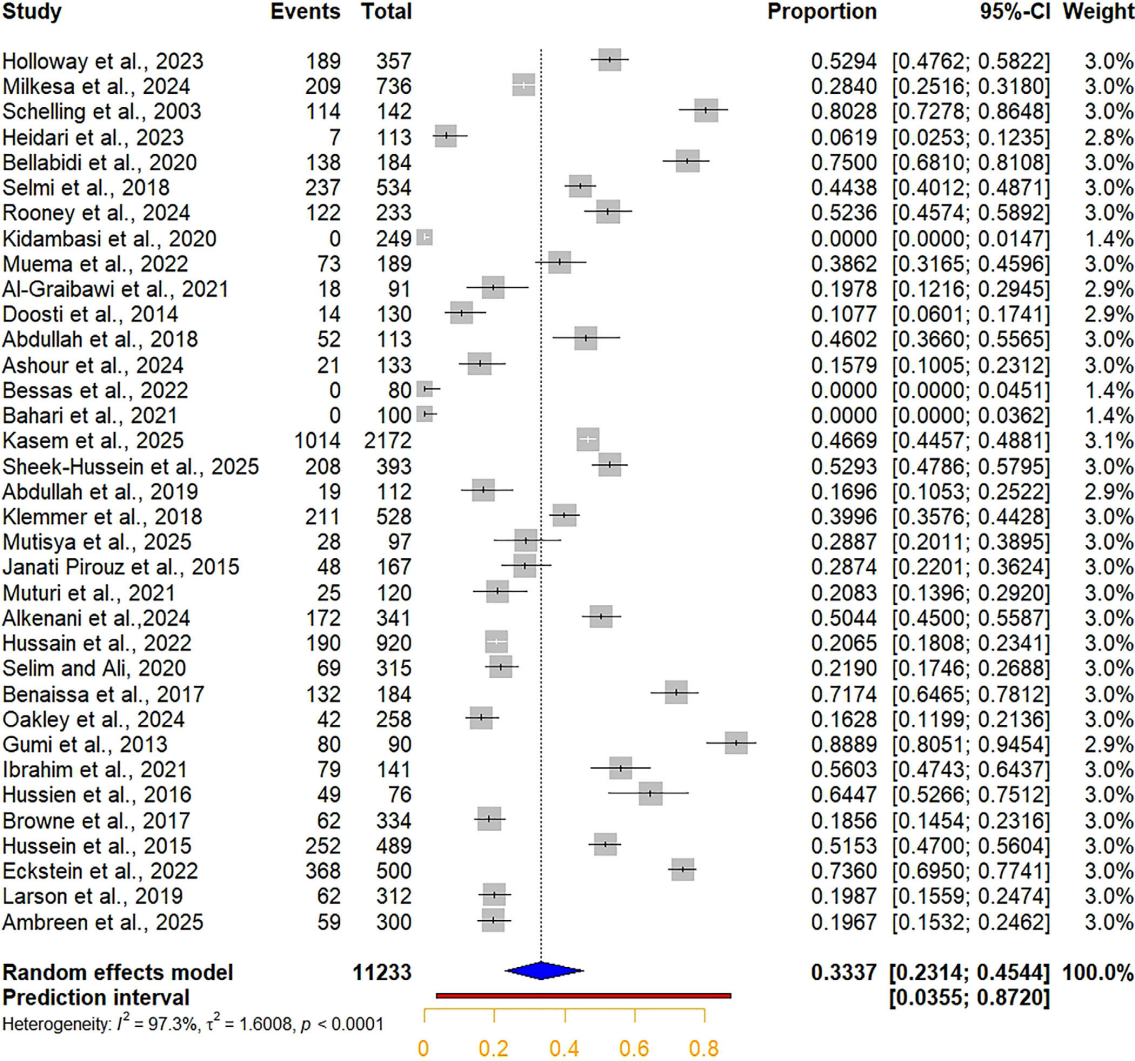
Pooled estimates of *C. burnetii* prevalence in camels

**TABLE 2:** Subgroup analysis of prevalence of *C. burnetii* in camels using different moderators.

Moderators		No studies	No tested	No positive	Pooled estimate (95% CI)	Heterogeneity				Subgroup differences
						Q	*I* ^2^ (%)	*τ* ^2^	*p*‐value	
Overall		35	11233	4363	33.37 [23.14; 45.44]	1278.56	97.3	1.6008	< 0.0001	
Continent	Africa	23	5660	2192	37.17 [23.00; 53.94]	863.03	97.5	1.8202	< 0.0001	*Q*_1_ =0.93; *p* = 0.3339
	Asia	12	5573	2171	26.87 [14.09; 45.15]	415.43	97.4	1.1495	< 0.0001	
Country	Algeria	3	448	270	31.30 [0.1; 99.95]	18.82	89.4	10.3007	< 0.0001	*Q*_12_ = 844.52 *p* < 0.0001
	Chad	1	142	114	80.28 [72.92; 86.03]	0.00	**—**	—	—	
	Egypt	5	1201	372	26.84 [13.44; 46.43]	65.48	93.9	0.4414	< 0.0001	
	Ethiopia	4	1225	410	48.02 [6.62; 92.33]	137.62	97.8	2.5292	< 0.0001	
	Iran	4	510	69	8.67 [0.77; 53.70]	33.50	91.0	1.6223	< 0.0001	
	Iraq	1	91	18	19.78 [12.83; 29.23]	0.00	—	—	—	
	Jordan	1	357	189	52.94 [47.75; 58.07]	0.00	—	—	—	
	Kenya	7	1534	372	23.42 [7.71; 52.83]	112.29	94.7	0.9299	< 0.0001	
	Pakistan	2	1220	249	20.41 [15.54; 26.33]	0.14	0.0	0	0.7131	
	Saudi Arabia	3	3002	1438	48.97 [42.30; 55.68]	4.75	57.9	0.0080	0.0931	
	Sudan	1	76	49	64.47 [53.15; 74.38]	0.0	—	—	—	
	Tunisia	2	1034	605	59.84 [0.05; 99.98]	87.52	98.9	0.7735	< 0.0001	
	UAE	1	393	208	52.93 [47.98; 57.82]	0.00	—	—	—	
Detection method	ELISA	26	9903	4191	44.06 [34.48; 54.09]	1043.87	97.6	0.9502	< 0.0001	*Q*_1_ = 11.42; *p* = 0.0007
	PCR	9	1330	172	7.76 [1.92; 26.54]	88.66	91.0	2.5373	< 0.0001	
Sample size	> 400	7	5879	2481	43.05 [27.36; 60.28]	427.22	98.6	0.5589	< 0.0001	*Q*_1_ = 1.73; *p* = 0.1885
	< 400	28	5354	1882	30.22 [18.53; 45.19]	842.51	96.8	2.0511	< 0.0001	
Publication year	2020 or before	16	3959	1539	39.43 [21.29; 61.04]	549.68	97.3	2.0891	< 0.0001	*Q*_1_ = 0.84; *p* = 0.3597
	After 2020	19	7274	2824	28.99 [17.87; 43.38]	728.41	97.5	1.1536	< 0.0001	

*Note*: Q, Cochran's heterogeneity statistic; *I* ^2^, percentage of total variation across studies attributable to between‐study heterogeneity; *τ* ^2^, estimated between‐study variance; *p*, *p*‐value for Cochran's Q test of heterogeneity.

Abbreviation: CI, confidence interval.

Subgroup analysis was performed to assess the contribution of continent, country, diagnostic test, sample size category and publication year for the prevalence of *C. burnetii* in camels (Table 2). According to continent, the pooled prevalence of *C. burnetii* appeared numerically higher in Africa (37.17%, 95%CI: [23.00; 53.94]) than in Asia (26.87%, 95% CI: [14.09; 45.15]). However, the difference between continents was not statistically significant difference observed (*Q*_1_ = 0.93; *p* = 0.3339) indicating no evidence of a true continental difference based on this subgroup analysis (Figure 3; Table 2). With regard to countries, despite five countries each reported single studies, the prevalence of Q fever ranges from 8.67% in Iran to 80.28 in Chad with a statistically significant difference between countries (*Q*_12_ = 844.52, *p* < 0.0001) (Table 2; Figure S1).

**FIGURE 3:**
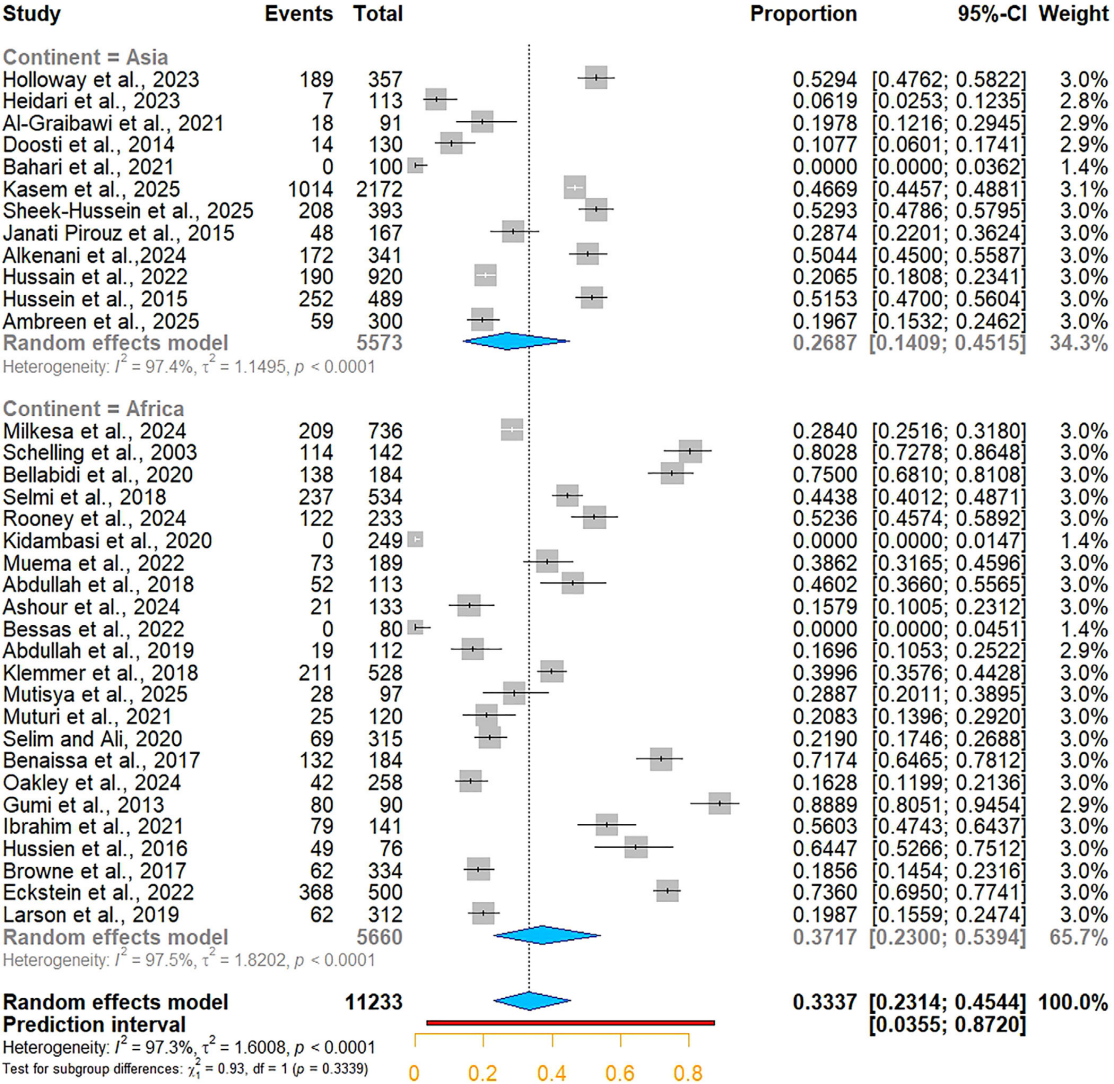
Forest plot showing subgroup analysis of *C. burnetii* prevalence in camels by continent.

Regarding diagnostic tests used, *C. burnetii* seroprevalence was higher in ELISA (44.06%, 95% CI: [34.48; 54.09]) than PCR (7.76%, 95% CI: [1.92; 26.54]) showing statistically significant difference between diagnostic tests (*Q*_1_ = 11.42, *p* = 0.0007) (Figure 4; Table 2).

**FIGURE 4:**
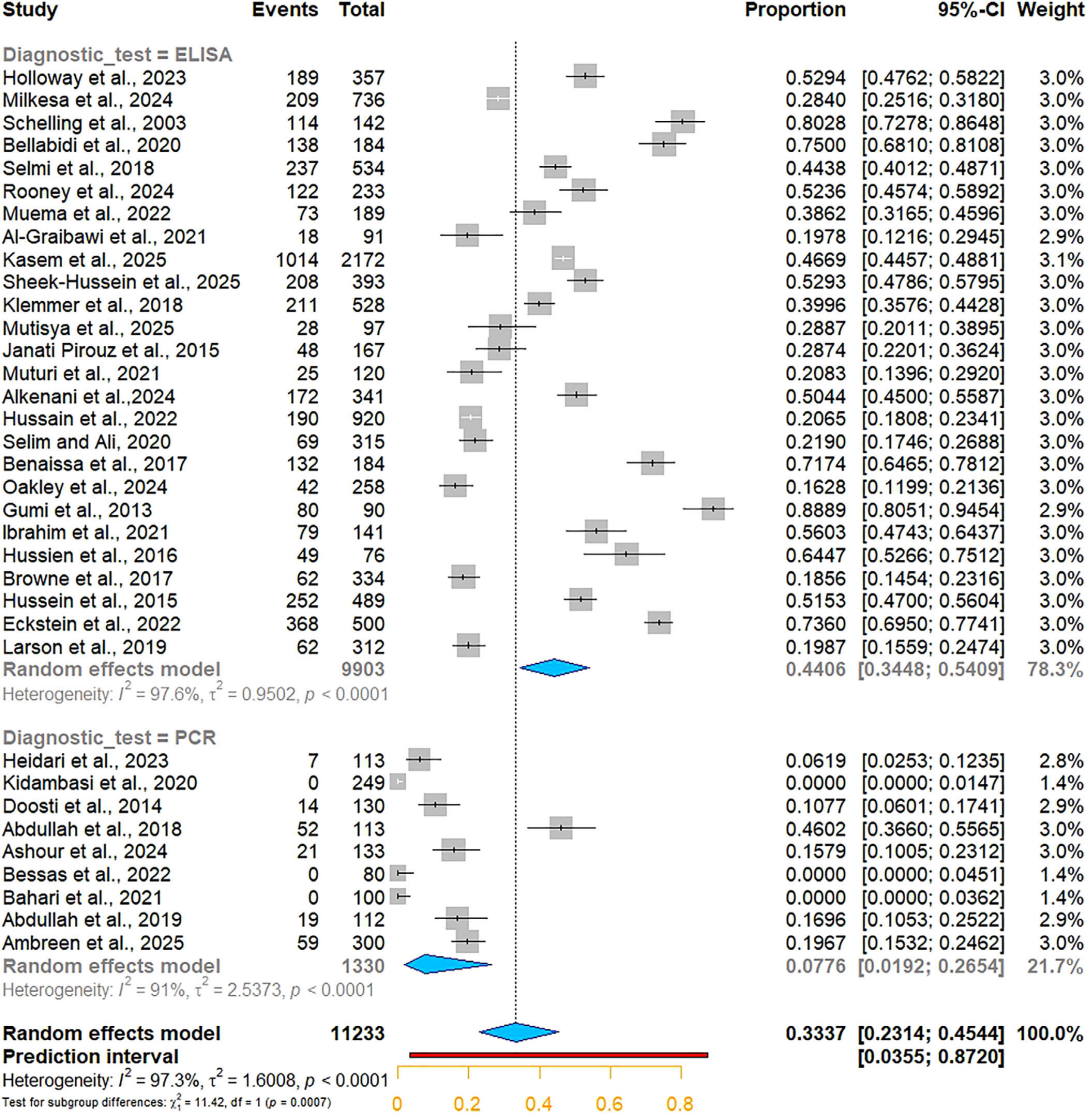
Forest plot showing subgroup analysis of *C. burnetii* prevalence in camels by diagnostic test.

The sample size was categorized into two groups (more than 400 and less than 400 samples). The pooled prevalence of *C. burnetii* was numerically higher in studies with more than 400 samples (43.05%, 95% CI: [27.36; 60.28]) than in those less than 400 samples (30.22%, 95% CI: [18.53; 45.19]); however, the difference was not statistically significant difference (*Q*_1_ = 1.73, *p* = 0.1885), suggesting no evidence of a true sample‐size‐related difference (Table 2; Figure S2).

According to publication year, the prevalence of *C. burnetii* was higher in studies published in 2020 and before (39.43%, 95% CI: [21.29; 61.04]) than those published after 2020 (28.99%, 95% CI: [17.87; 43.38]) with no statistical significant difference (*Q*_1_ = 0.84, *p* = 0.3597) (Table 2, Figure S3).

### 3.3 Assessment of Heterogeneity and Influence Analysis

To investigate the sources of the substantial between‐study heterogeneity observed, we employed both Galbraith radial plots and Baujat plots. The Galbraith radial plot (Figure 5) visualized the relationship between precision and standardized effect sizes, revealing significant dispersion with multiple studies falling outside the 95% confidence intervals, particularly identifying several imprecise studies with extreme negative effect sizes.

**FIGURE 5:**
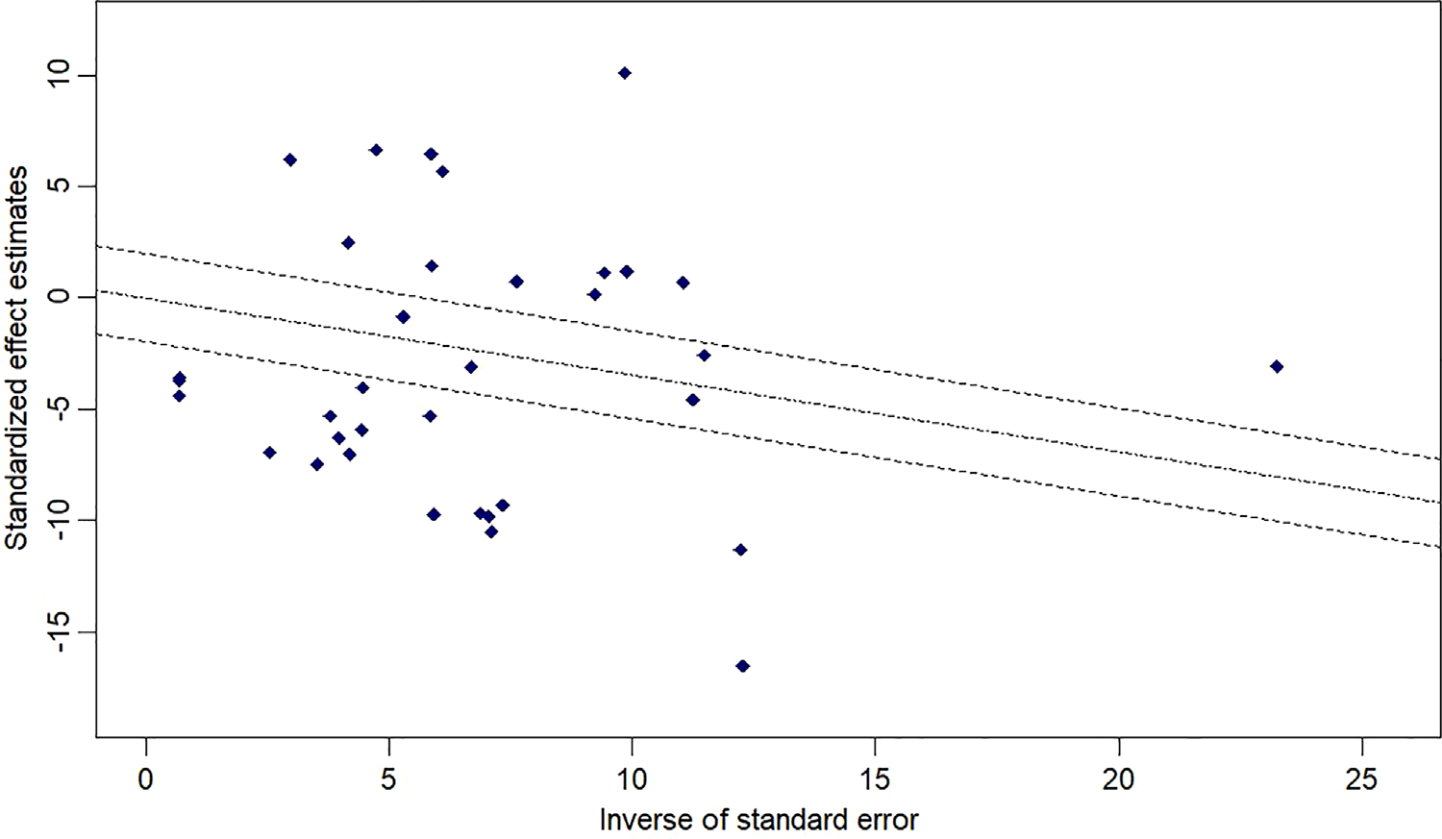
Galbraith (radial) plots.

Complementing this, the Baujat plot (Figure 6) was used to identify specific studies contributing disproportionately to the heterogeneity and the pooled prevalence estimate. The analysis highlighted distinct patterns of influence: Kidambasi et al. (2020) was identified as the most influential study on the overall pooled prevalence, despite a modest contribution to the global heterogeneity statistic. Conversely, Eckstein et al. (2022) was found to be the single largest contributor to the overall heterogeneity, although its individual influence on the pooled estimate was moderate. Other studies, such as Bahari et al. (2021) and Bessas et al. (2022), also showed considerable influence on the final result. These diagnostic plots suggest that the observed heterogeneity is driven by a subset of influential outliers rather than a systematic bias across all included studies.

**FIGURE 6:**
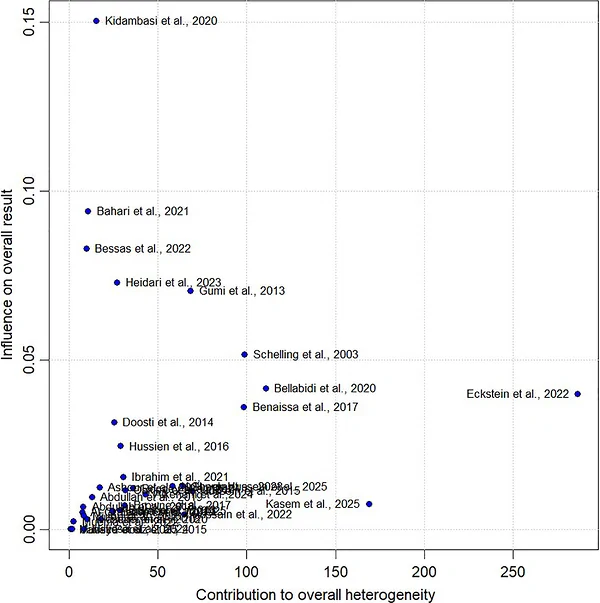
Baujat plot showing the contribution of each included study to overall heterogeneity (*x*‐axis) and its influence on the pooled effect estimate (*y*‐axis). Due to the number of included studies, some overlap of study labels is unavoidable.

To assess the robustness of the pooled estimate to the choice of transformation, a sensitivity analysis was performed using the Freeman–Tukey double‐arcsine transformation. The pooled prevalence was 33.90% (95% CI: 24.65; 43.81), compared with 33.37% (95% CI: 23.14; 45.44) using the logit transformation. The difference between the two estimates was small, indicating that the pooled estimate was robust to the choice of transformation.

A sensitivity analysis was conducted to evaluate the impact of influential studies and outliers. The sensitivity analysis by excluding one study at a time and recalculating the pooled prevalence showed no significant change in the pooled estimate ranging between 31.97% and 35.34%, as demonstrated in the leave‐one‐out meta‐analysis (Figure 7).

**FIGURE 7:**
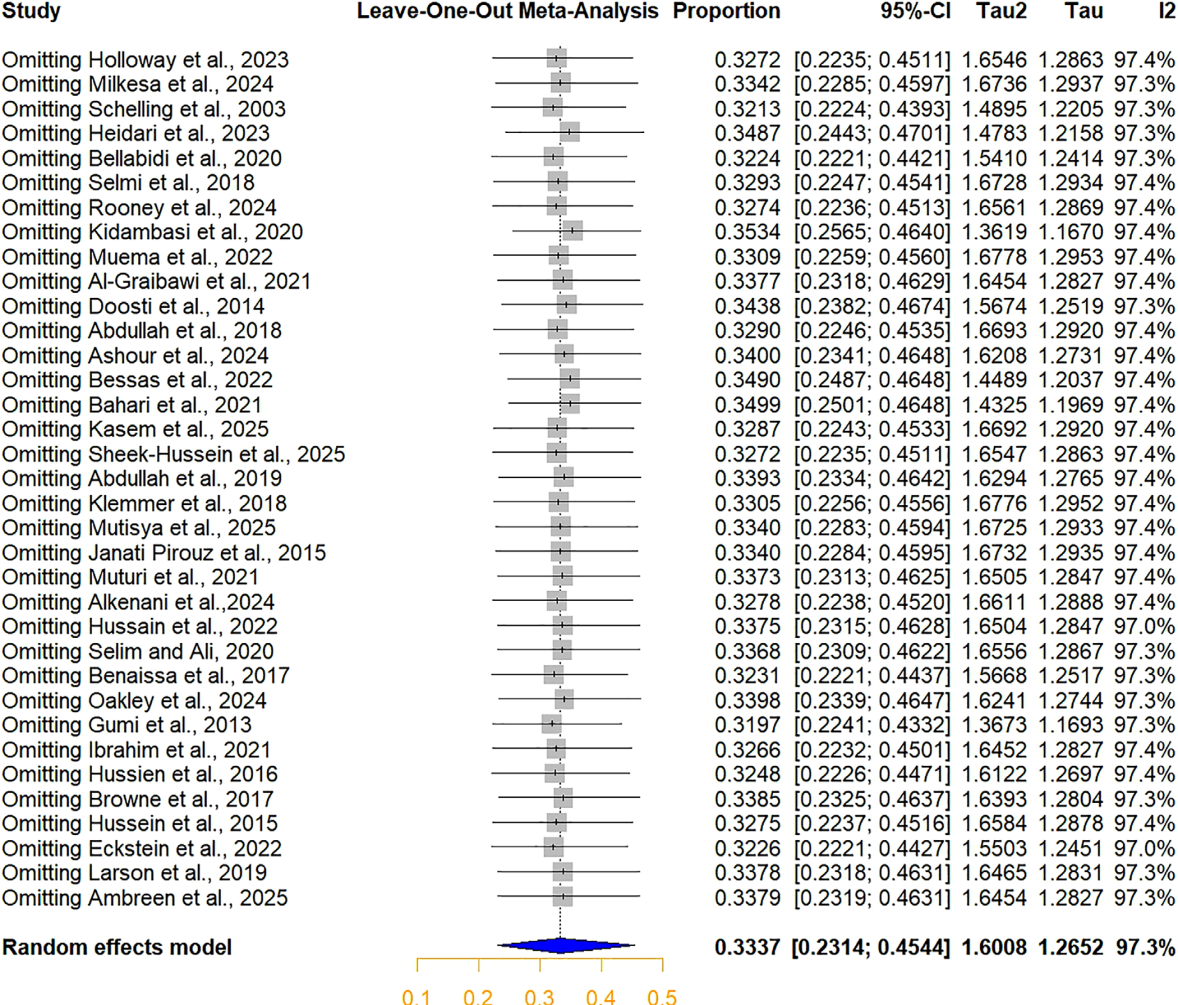
Leave one out meta‐analysis of *C. burnetii* prevalence studies in camels.

Excluding the single most influential study (Kidambasi et al. 2020) resulted in a marginal increase in pooled prevalence from 33.37% to 35.34% (Figure S4). Furthermore, excluding the four most influential outliers identified via diagnostic plots (Kidambasi et al. 2020; Eckstein et al. 2022; Bahari et al. 2021; Bessas et al. 2022) yielded a pooled prevalence of 36.96% (Figure S5). Despite these exclusions, the heterogeneity remained high (*I* ^2^ changed from 97.3% to 97.1%). Another sensitivity analysis was performed by excluding the four studies classified as moderate quality according to the JBI Critical Appraisal Checklist. The pooled prevalence changed only marginally from 33.37% (95% CI: [23.14; 45.44]) to 32.14% (95% CI: 22.35; 43.81). Likewise, the prediction interval changed only slightly from 3.55%–87.20% to 4.54%–82.52%, and between‐study heterogeneity remained virtually unchanged (I2 = 97.2%) (Figure S6). These findings indicate that the pooled prevalence estimate was robust to the exclusion of moderate‐quality studies.

### 3.4 Publication Bias

Publication bias was evaluated using both visual and statistical methods (Figure 8). The broadly symmetrical distribution of studies observed in the funnel plot suggests that there was no possibility of publication bias in *C. burnetii* studies in camels. This was further supported by non‐ significant *p*‐values of Begg's rank correlation test (*z* = −0.75, *p* = 0.4516) and Egger's regression test (*t* = −1.36, *p* = 0.1841). These findings suggest that there is no clear evidence of substantial publication bias in this meta‐analysis.

**FIGURE 8:**
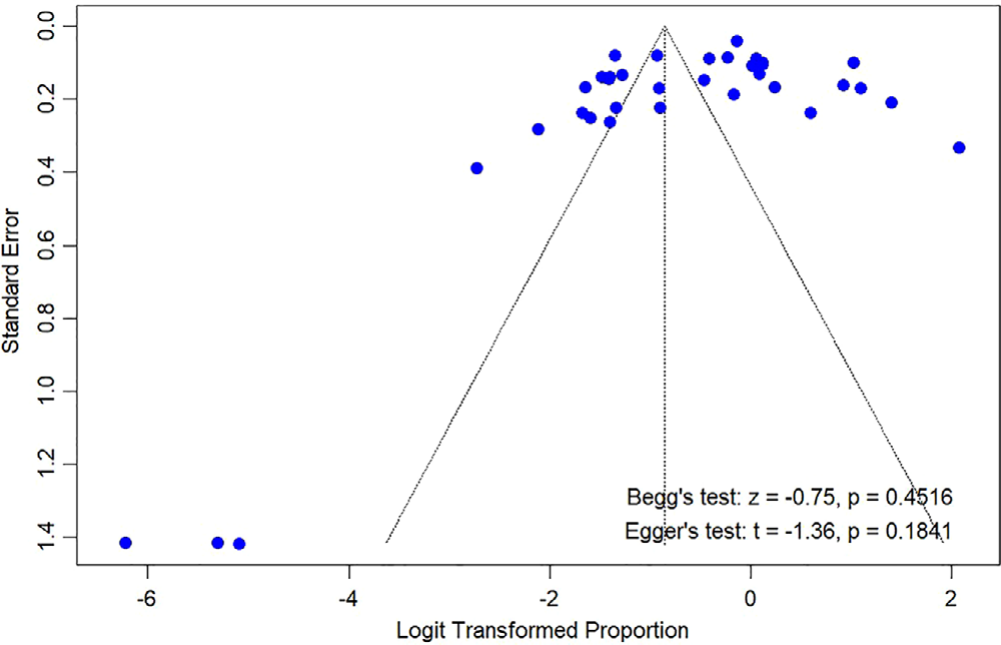
Funnel plot assessing potential publication bias among studies included in the meta‐analysis and the results of Begg's rank correlation test and Egger's regression test are embedded within the figure.

### 3.5 Association Between Prevalence of Q Fever and Sex of Camels and Tick Infestation Status

Among the 35 included studies in the meta‐analysis, 15 reported prevalence of Q fever with respect to sex of camels. Therefore, the risk factor analysis regarding sex showed the odds of female animals contracting Q fever were about 2.22 (95% CI: 1.53; 3.24) times higher than the odds in male camels (Figure 9).

**FIGURE 9:**
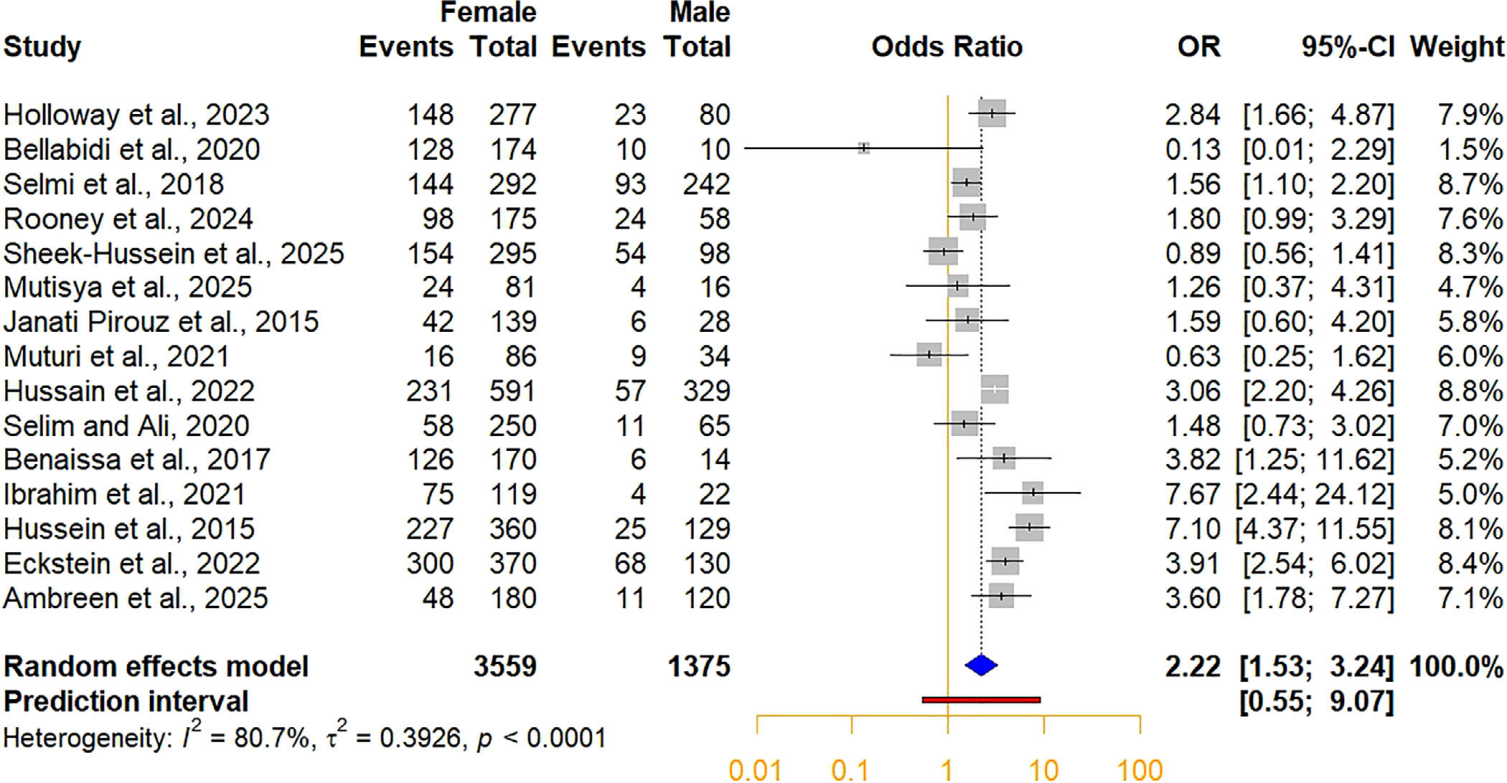
Forest plot of pooled odds ratio of sex and prevalence of Q fever in camels.

In addition, six studies reported information on tick infestation status in relation with Q fever prevalence and camels infested with ticks had significantly higher odds of *C. burnetii* infection compared with non‐infested animals, with an odds ratio of 2.09 (95% CI: 1.28; 3.41) (Figure 10).

**FIGURE 10:**
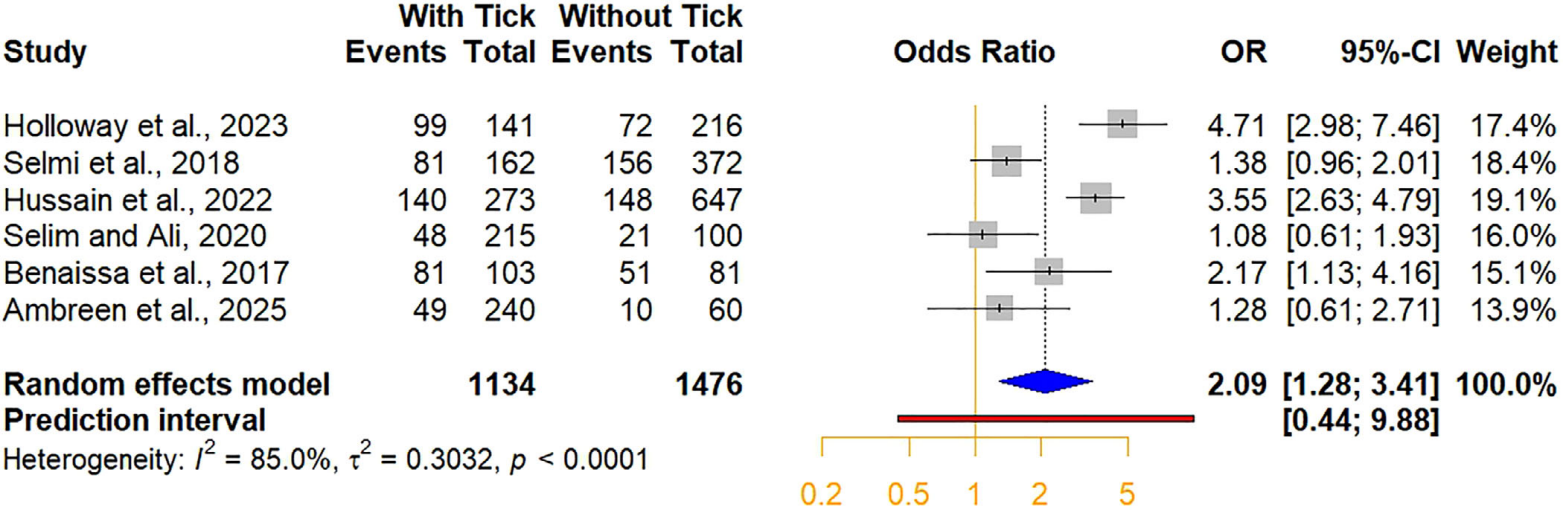
Forest plot of pooled odds ratio of tick infestation and prevalence of Q fever in camels.

## 4 Discussion

This study aimed to determine the overall prevalence of Q fever in camels and assess the heterogeneity between studies. In our systematic review and meta‐analysis, we estimated the prevalence of Q fever in 11,233 camels from 35 included studies in seven African and six Asian countries with a total of 4363 positive cases. The number of studies reporting Q fever in camels varied markedly by country, ranging from a maximum of seven studies in Kenya to only a single study each in Chad, Sudan, Iraq, Jordan and the United Arab Emirates, underscoring the overall insufficiency of available data across the continents and limited attention given to Q fever in camels in other parts of the globe. Moreover, more than half of the studies (54.3%) were carried out in the last five years.

The pooled prevalence of Q fever was 33.37%. Thus, more than one‐third of camels were exposed to *C. burnetii* infections. This finding was higher than 25.0% prevalence reported in camels in Eastern Mediterranean region (Ahmadinezhad et al. 2022). The pooled global prevalence of Q fever in camels should be interpreted with caution because substantial between‐study heterogeneity was observed (*I* ^2^ = 97.3%). This high heterogeneity likely reflects genuine differences in geographic regions, camel production and management systems, study populations, sampling strategies and diagnostic methods across the included studies. Furthermore, the wide 95% prediction interval (3.55%–87.20%) indicates that the prevalence of Q fever may vary considerably in future studies conducted under similar conditions, underscoring the marked epidemiological variability of the disease across different settings. Therefore, the pooled prevalence should be regarded as an overall summary of the available global evidence rather than a universally applicable estimate for all camel populations.

The quality‐based sensitivity analysis demonstrated that excluding the four moderate‐quality studies resulted in only minimal changes to the pooled prevalence, prediction interval and between‐study heterogeneity. This finding indicates that the overall estimate was robust and that the substantial heterogeneity observed was unlikely to be attributable to methodological quality alone. Instead, the remaining heterogeneity is more likely to reflect genuine differences among studies in terms of geographic region, study populations, husbandry and management practices, sampling strategies and diagnostic methods.

The pooled prevalence of Q fever observed in the present meta‐analysis indicates widespread exposure to *C. burnetii* among camel populations. This finding is biologically plausible given the epidemiological characteristics of the pathogen. *C. burnetii* is highly infectious, environmentally resilient and is shed in large quantities in placental tissues, birth fluids, milk, urine and faeces of infected animals, thereby contaminating the surrounding environment (Devaux et al. 2020; Kersh et al. 2013). Previous studies have suggested that pastoral production systems, herd mobility, communal grazing and watering practices, close contact with small ruminants and limited implementation of biosecurity measures may facilitate the transmission and maintenance of *C. burnetii* in camel populations (Browne et al. 2017; Bwatota et al. 2022; Holloway et al. 2023; Muema et al. 2022; Toledo‐Perona et al. 2024). However, these factors were not quantitatively synthesized in the present meta‐analysis because of inconsistent reporting across the included studies. Therefore, their contribution to the observed pooled prevalence should be interpreted as evidence from the broader literature rather than as findings of this study.

According to continents of the included studies, there was no significant difference between in the prevalence of Q fever in camels (37.17% in Africa and 26.87% in Asia). This data indicates that the prevalence of Q fever was high in both continents. The heterogeneity of the pooled prevalence in each continent is high, reflecting differences in the prevalence of Q fever within each continent or between studies within the same country. For example, in Africa, the prevalence was as high as 80.28% in Chad and as low as 23.42% in Kenya. The reported prevalence of Q fever in Algeria varied markedly, ranging from 75.0% (Bellabidi et al. 2020) to 0.0% (Bessas et al. 2022). Although it is difficult to compare the prevalence of Q fever between countries due to large differences in the number of included studies, total number of camels tested, and differences in technical methods of diagnosis, the prevalence ranges from 8.67% in Iran to 80.28% in Chad. These findings showed that the prevalence of Q fever in camels was high and variations in prevalence among countries can be influenced by a complex interplay of ecological, social, cultural, behavioural and economic factors, in addition to differences in the burden of animal diseases.

In this meta‐analysis, 26 studies employed ELISA and 9 studies employed PCR as diagnostic method for *C. burnetii* in camels. Across these studies, the pooled seroprevalence based on ELISA was substantially higher (44.06%) than the pooled molecular prevalence by PCR (7.76%). This discrepancy likely reflects differences in the underlying sample sizes, with only 1330 samples tested by PCR compared with 9903 samples assessed using ELISA. In addition, PCR detects *C. burnetii* DNA, indicating active infection or bacterial presence at the time of sampling; however, the bacterium may be transient or present at low levels, resulting in lower detection rates, particularly outside acute infection phases. In contrast, ELISA detect host antibodies against *C. burnetii*, reflecting both recent and past exposures (Plummer et al. 2018). Consequently, differences in prevalence estimates between diagnostic methods should be interpreted in light of the distinct epidemiological information provided by each assay.

In this meta‐analysis, the prevalence of Q fever in camels is higher in female animals than males and the odds of infection in female camels was more than twice as compared to males. This finding was in agreement with another meta‐analysis on global and regional seroprevalence of coxiellosis in small ruminants (Ahaduzzaman and Reza 2024) and prevalence of Q fever in the Eastern Mediterranean region (Ahmadinezhad et al. 2022), where female animals are more susceptible to *C. burnetii* than male animals. Our finding was in line with the reports of several individual studies in small ruminants (Esmaeili et al. 2013; Sakhaee and Khalili 2010; Siengsanan‐Lamont et al. 2023; Zahid et al. 2016) and domestic ruminants (Abakar et al. 2014; Troupin et al. 2022). This could be attributed to the association of *C. burnetii* with female reproductive tissues and secretions. *C. burnetii*’s strong affinity for reproductive tissues, in particular for placental trophoblasts, at which the pathogen replicates to extraordinary titers creating a tissue‐specific amplification niche absent in males (Howard and Omsland 2020; Zarza et al. 2021). This bacterium can be shed during subsequent births and lactation. Moreover, female animals are often kept in production systems for extended periods for breeding and milk production, leading to prolonged exposure to contaminated environments. These might be indicators of the critical epidemiological role of females in maintaining *C. burnetii* circulation within camel populations, wherein breeding‐age females serve as the primary amplification reservoirs and environmental shedders. Understanding this sex‐stratified susceptibility is essential for targeted surveillance and intervention strategies in pastoral camel systems, as reproductive management practices influencing female pregnancy rates and parturition timing directly impact herd‐level *C. burnetii* transmission dynamics and zoonotic spillover risk to human populations.

The prevalence of Q fever was significantly higher in camels infested with ticks compared with those without tick infestation. Ticks are recognized reservoirs and potential vectors of *C. burnetii*, and *C. burnetii* DNA has been detected in several ixodid tick species commonly parasitizing camels and other domestic animals (Abdullah et al. 2018; Ali et al. 2023; Ashour et al. 2024; Celina and Cerný 2022; Mediannikov et al. 2010; Nooma et al. 2025; Rooney et al. 2024; Sulyok et al. 2014), suggesting their involvement in maintaining and cycling the pathogen within camel herds. Field studies in camels and other ruminants have consistently reported a positive association between tick infestation and *C. burnetii* positivity, with the presence or high burdens of ticks increasing the odds of infection (Benaissa et al. 2017; Hussain et al. 2022). In extensive pastoral systems, where acaricide use and tick control are limited, sustained high tick burdens likely enhance repeated exposure to infected ticks, thereby increasing infection pressure and contributing to the elevated Q fever prevalence.

## 5 Conclusion

This systematic review and meta‐analysis provide a comprehensive assessment of *C. burnetii* prevalence in camels globally, revealing a pooled prevalence of 33.37% across Africa and Asia. This substantial burden underscores Q fever as a significant zoonotic concern in camel‐producing regions and highlights the role of camels as important reservoirs of *C. burnetii*. Despite the evidence of substantial Q fever burden in camels, notable gaps remain in the global epidemiological landscape. The absence of published prevalence data from the other parts of the world limits our ability to assess whether camel Q fever represents a truly global zoonotic threat or remains primarily endemic to Africa and Asia. These findings highlight substantial heterogeneity in the epidemiology of Q fever among camel populations and underscore the importance of strengthening surveillance, standardized diagnostic approaches and targeted epidemiological investigations to better characterize the burden and distribution of *C. burnetii* infection in camels.

### 5.1 Limitations of the Study

This systematic review and meta‐analysis have several limitations. Only studies published in English with full‐text available were included; therefore, studies published in other languages and those not open access are not reflected in the analysis. In addition, the literature search was restricted to indexed electronic databases, and locally published non‐indexed journals and studies identified through hand‐searching were not included, which may have resulted in the omission of relevant studies. The pooled estimates should be interpreted cautiously because of the very high heterogeneity across studies, which suggests substantial variation in study populations, settings, sampling strategies and epidemiological contexts. The country‐specific subgroup analysis should also be interpreted with caution because several countries were represented by only a single study. Consequently, these estimates reflect the available published evidence rather than the true national prevalence of Q fever in camels and should not be used to make direct comparisons between countries. Additional epidemiological studies with broader geographic coverage are needed to generate more representative national prevalence estimates. Additional epidemiological studies with broader geographic coverage are needed to generate more representative national prevalence estimates. Furthermore, the included studies used different diagnostic approaches, which may have affected the detection of *C. burnetii* and contributed to between‐study variability. Differences in test sensitivity and specificity can influence prevalence estimates and limit direct comparability across studies. Although the search was not restricted by continent, eligible studies were identified only from Africa and Asia; thus, the evidence base may not fully represent the global distribution of *C. burnetii* and limits the generalizability of the findings to other regions. Although publication bias was assessed using funnel plots, Egger's regression test and Begg's rank correlation test, these approaches have recognized limitations in meta‐analyses of prevalence data. The asymmetry of funnel plots and the results of statistical tests may be influenced by substantial between‐study heterogeneity, the bounded nature of proportion data and differences in study size, diagnostic methods and study populations rather than publication bias alone. Nevertheless, funnel plots together with Egger's and Begg's tests remain widely accepted tools for the exploratory assessment of potential small‐study effects and publication bias and were used to provide complementary evidence rather than definitive proof of publication bias.

## Author Contributions

*Conceptualization*: SAK. *Methodology*: SAK, AMW, MKB, YGA. *Formal analysis*: SAK. *Data curation*: SAK, SM, PWA, AMW, MTM, SZA, YAE, KS, AMM, YGA. *Visualization*: SAK. *Validation*: AMW, MKB, SGE, YGA. *Writing – original draft*: SAK. *Writing – review and editing*: SM, PWA, AMW, MKB, MTM, SZA, YAE, KS, SGE, AMM, YGA.

## Funding

The authors have nothing to report.

## Ethics Statement

The authors have nothing to report.

## Conflicts of Interest

The authors declare no conflicts of interest.

## Supporting information




**Supporting File 1**: vms371275‐sup‐0001‐S1‐S6 R2.docx

**Supporting File 2**: vms371275‐sup‐0002‐S1.docx

## Data Availability

All the data used for analysis are available from the corresponding author upon reasonable request.
