# Supplementary material for: Global Prevalence of Q Fever in Camels: A Systematic Review and Meta‐Analysis of Studies From Africa and Asia
Source: Vet Med Sci. 2026 Oct 21;12(6):e71275. doi: 10.1002/vms3.71275 (PMC13685673; doi:10.1002/vms3.71275)
Supplement: Supplementary file 1 [file VMS3-12-e71275-s001.docx]

**Fig. S1.** Forest plot showing subgroup analysis of Q fever prevalence in camels by country

**Fig. S2.** Forest plot showing subgroup analysis of Q fever prevalence in camels by sample size

**Fig. S3.** Forest plot showing subgroup analysis of Q fever prevalence in camels by study period

**Fig. S4** Pooled prevalence of Q fever in camels after excluding the single most influential study (after excluding Kidambasi et al., 2020)

**Fig. S5** Pooled prevalence of Q fever in camels after excluding the four most influential outliers (after excluding Kidambasi et al., 2020; Eckstein et al., 2022; Bahari et al., 2021; Bessas et al., 2022)

**Fig. S6** Forest plot of the sensitivity analysis excluding the four studies classified as moderate quality according to the Joanna Briggs Institute (JBI) Critical Appraisal Checklist. The pooled prevalence estimate, 95% confidence interval, prediction interval, and between-study heterogeneity remained largely unchanged compared with the primary analysis, demonstrating the robustness of the overall findings.
