# Supplementary material for: Global Prevalence of Q Fever in Camels: A Systematic Review and Meta‐Analysis of Studies From Africa and Asia
Source: Vet Med Sci. 2026 Oct 21;12(6):e71275. doi: 10.1002/vms3.71275 (PMC13685673; doi:10.1002/vms3.71275)
Supplement: Supplementary file 2 [file VMS3-12-e71275-s002.docx]

**Table S1**. JBI quality assessment of the included studies

| Author | Was the sample frame appropriate to address the target population | Were study participants sampled in an appropriate way? | Was the sample size adequate? | Were the study subjects and the setting described in detail? | Was the data analysis conducted with sufficient coverage of the identified sample? | Were valid methods used for the identification of the condition? | Was the condition measured in a standard, reliable way for all participants? | Was there appropriate statistical analysis? | Was the response rate adequate, and if not, was the low response rate managed appropriately? |
| --- | --- | --- | --- | --- | --- | --- | --- | --- | --- |
| Holloway et al., 2023 | Yes | Yes | Yes | Yes | Yes | Yes | Yes | Yes | Yes |
| Milkesa et al., 2024 | Yes | Yes | Yes | Yes | Yes | Yes | Yes | Yes | Yes |
| Schelling et al., 2003 | No | Yes | No | Yes | Yes | Yes | Yes | No | Yes |
| Heidari et al., 2023 | Yes | No | Yes | Yes | Yes | Yes | Yes | Yes | No |
| Bellabidi et al., 2020 | Yes | No | Yes | Yes | Yes | Yes | Yes | Yes | No |
| Selmi et al., 2018 | Yes | No | Yes | Yes | Yes | Yes | Yes | Yes | No |
| Rooney et al., 2024 | Yes | Yes | Yes | Yes | Yes | Yes | Yes | Yes | Yes |
| Kidambasi et al., 2020 | Yes | Yes | Yes | Yes | Yes | Yes | Yes | Yes | Yes |
| Muema et al., 2022 | Yes | Yes | Yes | Yes | Yes | Yes | Yes | Yes | Yes |
| Al-Graibawi et al., 2021 | Yes | No | No | Yes | Yes | Yes | Yes | Yes | Yes |
| Doosti et al., 2014 | Yes | No | Yes | No | Yes | Yes | Yes | No | Yes |
| Abdullah et al., 2018 | Yes | Yes | Yes | Yes | Yes | Yes | Yes | No | Yes |
| Ashour et al., 2024 | Yes | Yes | Yes | Yes | Yes | Yes | Yes | No | Yes |
| Bessas et al., 2022 | Yes | No | Yes | Yes | Yes | Yes | Yes | No | Yes |
| Bahari et al., 2021 | Yes | Yes | No | Yes | Yes | Yes | Yes | Yes | Yes |
| Kasem et al., 2025 | Yes | Yes | Yes | Yes | Yes | Yes | Yes | Yes | Yes |
| Sheek-Hussein et al., 2025 | Yes | Yes | Yes | Yes | Yes | Yes | Yes | Yes | Yes |
| Abdullah et al., 2019 | No | Yes | No | Yes | Yes | Yes | Yes | Yes | Yes |
| Klemmer et al., 2018 | Yes | Yes | Yes | Yes | Yes | Yes | Yes | Yes | Yes |
| Mutisya et al., 2025 | Yes | Yes | Yes | Yes | Yes | Yes | Yes | Yes | Yes |
| Janati Pirouz et al., 2015 | Yes | Yes | Yes | Yes | Yes | Yes | Yes | Yes | Yes |
| Muturi et al., 2021 | Yes | Yes | Yes | Yes | Yes | Yes | Yes | Yes | Yes |
| Alkenani et al., 2024 | Yes | Yes | Yes | Yes | Yes | Yes | Yes | Yes | Yes |
| Hussain et al., 2022 | Yes | Yes | Yes | Yes | Yes | Yes | Yes | Yes | Yes |
| Selim and Ali, 2020 | Yes | Yes | Yes | Yes | Yes | Yes | Yes | Yes | Yes |
| Benaissa et al., 2017 | Yes | Yes | Yes | Yes | Yes | Yes | Yes | Yes | Yes |
| Oakley et al., 2024 | Yes | Yes | Yes | Yes | Yes | Yes | Yes | Yes | Yes |
| Gumi et al., 2013 | Yes | Yes | No | Yes | Yes | Yes | Yes | No | No |
| Ibrahim et al., 2021 | Yes | Yes | Yes | Yes | Yes | Yes | Yes | Yes | Yes |
| Hussien et al., 2016 | Yes | Yes | Yes | Yes | Yes | Yes | Yes | No | Yes |
| Browne et al., 2017 | Yes | Yes | Yes | Yes | Yes | Yes | Yes | Yes | Yes |
| Hussein et al., 2015 | Yes | Yes | Yes | Yes | Yes | Yes | Yes | Yes | Yes |
| Eckstein et al., 2022 | Yes | Yes | Yes | Yes | Yes | Yes | Yes | Yes | Yes |
| Larson et al., 2019 | Yes | Yes | Yes | Yes | Yes | Yes | Yes | Yes | Yes |
| Ambreen et al., 2025 | No | No | No | Yes | Yes | Yes | Yes | Yes | Yes |
